# Assessing the Potential of Caprine Collagen Type I in the Development of Medical Devices

**DOI:** 10.1021/acs.biomac.5c00309

**Published:** 2025-09-01

**Authors:** Ignacio Sallent, Arely Leon Lopez, Gabriel Aguirre-Álvarez, Dimitrios I. Zeugolis

**Affiliations:** † Regenerative, Modular & Developmental Engineering Laboratory (REMODEL) and CÚRAM Research Ireland Centre for Medical Devices, 8799 University of Galway, Galway H91 TK33, Ireland; ‡ TecNM Campus Venustiano Carranza, Puebla 73049, Mexico; § Agricultural Sciences Institute, Autonomous University of Hidalgo State, Hidalgo 43775, Mexico; ∥ Regenerative, Modular & Developmental Engineering Laboratory (REMODEL), Charles Institute of Dermatology, Conway Institute of Biomolecular & Biomedical Research and School of Mechanical & Materials Engineering, 8797 University College Dublin (UCD), Dublin D04 V1W8, Ireland

## Abstract

We isolated collagen I from caprine, porcine, and bovine skin and tendon tissues and assessed their purity and chemical properties. We fabricated non-cross-linked and cross-linked scaffolds from each collagen I preparation and assessed their physicochemical and biological properties. Purity and chemical analyses did not reveal any notable differences between the groups. Fibril diameter analysis revealed that the non-cross-linked and cross-linked porcine, caprine, and bovine preparations resulted in scaffolds with the thickest, intermediate, and thinnest fibrils. Among the non-cross-linked scaffolds, no significant differences were observed in stress at break between the groups and the caprine scaffolds had significantly lower and higher strain at break and Young’s modulus than the bovine and the porcine scaffolds. Cross-linking significantly increased stress at break of all scaffolds, strain at break of the caprine scaffolds, and Young’s modulus of the bovine and porcine scaffolds. Biological analysis did not reveal any noteworthy differences between the groups.

## Graphical abstract

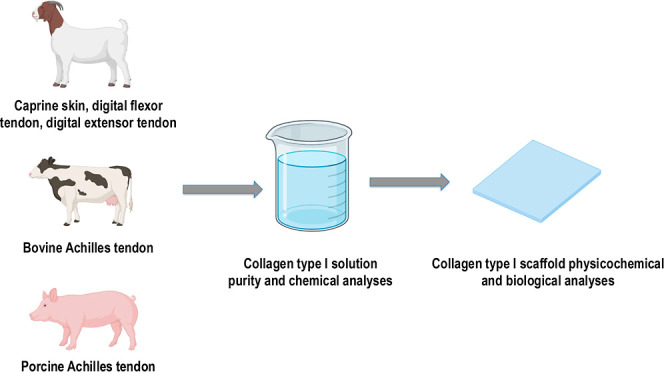

## 1 Introduction

Collagen type I is the most abundant protein in human body and plays a pivotal role in maintaining the structural and biological integrity of tissues.^1^ Through intricate interactions with other extracellular matrix (ECM) components, collagen type I gives rise to hierarchically organized supramolecular structures that possess unique morphological and mechanical properties.^2^ Considering its low immunogenicity and innate cell-recognition motifs, collagen type I is the building block of manyfold medical devices for a diverse range of clinical indications.^3^ Recent advancements in collagen type I recombinant expression^4^ and peptide synthesis^5^ have circumvented issues linked to animal-derived collagen type I, such as batch-to-batch variability and the potential risk of interspecies disease transmission. Nevertheless, these techniques continue to face important biological (e.g., insufficient post-translational modifications that are associated with functionality and stability issues) and commercial (e.g., high manufacturing costs, low yields) challenges.^5–7^ To date, collagen type I from mammalian tissues remains the primary source of collagen to meet market demands.

Despite the global prevalence of bovine and porcine tissues for collagen type I sourcing, religious constraints and concerns over disease transmission (i.e., bovine spongiform encephalopathy and foot-and-mouth disease, respectively) restrain their use and underscore the imperative need for alternative collagen type I sources.^8,9^ Although tissues from aquatic,^10^ avian,^11^ and amphibian^12^ species have shown promise, mammalian in origin collagen type I remains the preferred choice for clinical applications due to its homology with human collagen.^13^ Amidst these considerations, caprine tissues emerge as a potential collagen type I source, considering the reduced risk of zoonotic disease transmission and the lack of religious constraints.^14^ Further, considering the established contribution of animal antibiotic usage to antibiotic resistance in humans,^15^ one should note that caprine breeding involves significantly lower antibiotic use than porcine and bovine breeding^16^ and therefore carries lower responsibility for antibiotic resistance to humans.

In this context, we hypothesized that caprine collagen type I shares comparable physicochemical and biological properties with collagen type I from bovine Achilles tendon (BAT) and porcine Achilles tendon (PAT). Considering that collagen preparations maintain memory of the tissue from which the collagen is extracted from,^17–20^ we extracted, using the acid/pepsin process that is known to increase yield and reduce collagen’s antigenicity and immunogenicity,^21–23^ collagen type I from three different tissues (caprine skin (CS), caprine digital flexor tendon (CDFT), and caprine digital extensor tendon (CDET)) and assessed their purity. Subsequently, collagen films were produced from all different collagen preparations, cross-linked with 4-arm poly­(ethylene glycol) succinimidyl glutarate (4SP), a well-established cytocompatible collagen cross-linking agent,^24–28^ and their physicochemical and biological properties were assessed.

## 2 Materials and Methods

### 2.1 Materials

Bovine (∼26 months old animals), caprine (∼12 months old animals), and porcine (∼6 months old animals) tissues were obtained from a local slaughterhouse and transferred to our laboratory in ice. The 4SP was purchased from JenKem Technology (United States of America). Cell culture consumables were purchased from Sarstedt (Ireland). Chemicals, reagents, laboratory consumables, and cell culture media were purchased from Sigma-Aldrich (Ireland), unless otherwise specified.

### 2.2 Collagen Type I Extraction

Collagen type I was isolated from bovine and porcine Achilles tendons as well as from caprine skin, digital flexor, and extensor tendons. Caprine skin fur was sheared with an electric razor and the skin tissues were cut in ∼1 cm^3^ pieces with a sterile scalpel, washed three times for 2 h each with phosphate buffered saline (PBS), and frozen. The tendon tissues were physically separated from the fascia, cut in ∼1 cm^3^ pieces with a sterile scalpel, washed three times for 2 h each with PBS, and frozen. Collagen type I was extracted based on established protocols.^22,23^ Briefly, frozen tendon and skin tissue pieces were cryomilled (Freezer/Mill 6870, SPEX SamplePrep, United States of America), dissolved in 1.0 M acetic acid (100 mL per g of tissue), and pepsin digested (40 U per mg of tissue) at 4 °C for 72 h under continuous mechanical stirring. Nonsoluble matter was removed by centrifugation (21,000*g*) and 100 μm filtration. The final collagen solutions, obtained after 0.9 M NaCl precipitation, solubilization in 1.0 M acetic acid, and dialysis (molecular weight cut off: 8000 Da) against 1.0 mM acetic acid, were freeze-dried (FreeZone, Labconco, United States of America) and stored at −20 °C until use.

### 2.3 Evaluation of Collagen Preparation Purity and Chemical Properties

The purity and chemical properties of the collagen preparations were assessed based on established protocols.^29,30^ Briefly, freeze-dried collagens were dissolved in 0.5 M acetic acid at a concentration of 0.25 mg/mL, mixed with running buffer, and heated at 95 °C for 5 min. Each collagen sample was loaded in a sodium dodecyl sulfate polyacrylamide gel (3% stacking gel, 5% separation gel) and run in a Mini-PROTEAN Tetra Electrophoresis System (Bio-Rad Laboratories, United Kingdom) by applying a difference in the electric potential of 50 mV for the initial 30 min and 120 mV the following 60 min. The protein in the gel was visualized by means of silver staining (SilverQuest, Invitrogen, Thermo Fisher Scientific, Ireland) according to the manufacturer’s protocol. Freeze-dried collagens were analyzed with an IRSspirit Fourier transform infrared (FTIR) spectrophotometer (Shimadzu, Japan) and an attenuated total reflection attachment (Specac, United Kingdom) was utilized to analyze the samples directly in the solid state. The triple helix integrity ratio was determined from FTIR spectra by dividing the absorbance at the amide III band (1236 cm^–1^), sensitive to the collagen triple helical structure, by the absorbance at 1450 cm^–1^, used as a structural reference band.

### 2.4 Collagen Film Fabrication

Collagen scaffolds were fabricated as per established protocols.^31,32^ Briefly, 6 mg/mL collagen type I was mixed with 10× PBS in a 10:1 ratio. Using a two-syringe connected via a two-way stopcock device setup, the collagen solution was mixed with PBS (−4SP) or 1 mM 4SP (+4SP) for only 10 s to prevent premature gelation. The resulting mixture was poured onto silicon molds and placed in a laminar flow hood for 24 h to allow for solvent evaporation. The collagen films were peeled off from the molds and preserved in a dry atmosphere at 4 °C until further use.

### 2.5 Evaluation of Collagen Film Physicochemical Properties

Macroscopic and microscopic topographical analyses were performed by direct observation and by Atomic Force Microscopy (AFM) using a Veeco Dimension 3100 (Veeco, Netherlands) followed by Gwyddion software (Czech Metrology Institute, Czech Republic) analysis. Mean surface roughness was calculated by root-mean-square, and the fibril size diameter was assessed manually for a total of 40 fibrils from 4 different scaffold locations.

Free amines were quantified by means of the 2,4,6-trinitrobenzenesulfonic acid (TNBSA, Thermo Fisher Scientific, Ireland) assay, as has been described previously.^29^ Briefly, ∼2.5 mg of each collagen scaffold were mixed with 0.1 M sodium bicarbonate and 0.01% (wt/vol) TNBSA and incubated at 37 °C for 2 h under constant orbital agitation. The reaction was stopped by the addition of 10% (wt/vol) sodium dodecyl sulfate and 1 M HCl. Subsequently, the samples were hydrolyzed at 95 °C for 30 min. Absorbance was measured at 335 nm (Varioskan Flash Multimode Reader, Thermo Fisher Scientific, Ireland). Glycine, at different concentrations, was used to generate a standard curve. Results are displayed as % of free amines.

The mechanical properties of the collagen scaffolds were assessed using a Z005 (Zwich/Roell, Germany) universal testing machine, as has been previously described.^31^ Briefly, films were cut in a uniform rectangular shape (5 cm length and 1 cm width) and immersed in 1× PBS overnight. The hydrated scaffolds were blotted with filter paper to remove surface-bound PBS and their thickness was measured with a RS PRO micrometer digital calliper (Radionics, Ireland). Samples were affixed to the clamps by the extremities (3 cm distance between the clamps, 2 cm scaffold length between the clamps), preloaded to 0.01 N, and stretched uniaxially at a rate of 10 mm/min until failure. The stress was measured with a 10 N load cell. The following parameters were calculated: stress at break, strain at break, and Young’s modulus. Stress at break was calculated by dividing the force at failure by the initial area. Strain at break was determined as the increase in length from the start to failure. Young’s modulus was calculated as the slope of the linear region of the stress–strain curve prior to the yield point.

Resistance to enzymatic degradation was assessed following a modified version of a previously established protocol.^33^ In brief, −4SP and +4SP collagen scaffolds were incubated for 2 h in 0.1 M Tris-HCl and 5 mM CaCl_2_ at pH 7.4 buffer and then they were exposed to bacterial collagenase type II (MMP-8, 10 U/ml, Thermo Fisher Scientific, Ireland), prepared in the same buffer, for 12, 24, 36, and 48 h at 37 °C. The amount of degraded collagen at different time points was determined using the Pierce BCA Protein Assay Kit (Thermo Fisher Scientific, Ireland).

### 2.6 Evaluation of Collagen Film Biological Properties Using Human Skin Fibroblasts and Human Macrophages

Under sterile conditions, collagen scaffolds were cut in circular shapes, placed in 24-well plates, and fixed to the bottom using sterilized rubber O-ring. Scaffolds were immersed in 70% ethanol for 30 min, washed three times (5 min each) with 1× PBS, and incubated with high-glucose Dulbecco’s modified Eagle’s medium (DMEM), supplemented with 1% penicillin/streptomycin and 10% fetal bovine serum for 2 h. Human skin fibroblasts (WS1, American Type Culture Collection, United Kingdom) were seeded onto the scaffolds at a density of 20 × 10^3^ cell/cm^2^ and left to attach at 37 °C and 5% CO_2_. After 24 h, the media were replaced with new media and the cells were cultured for 3, 5, and 7 days with medium change every 2 days. Metabolic activity was assessed with the alamarBlue assay (Invitrogen, Thermo Fisher Scientific, Ireland), as per manufacturer’s guidelines, and is expressed as % reduction of alamarBlue normalized to the activity of cells cultured on tissue culture plastic (TCP) at each time point. To account for potential interference of collagen scaffolds with the alamarBlue assay, a negative control consisting of cell-free collagen scaffolds was included in every experiment. The average absorbance of these controls was subtracted from the corresponding values of the experimental samples to ensure an accurate measurement of cell metabolic activity. Cell viability was assessed using the ethidium homodimer-1 and calcein AM method.^31^ Briefly, at each time point, scaffolds were washed with 1× PBS and incubated with 2 mM ethidium homodimer-1 and 4 mM calcein AM diluted in 1× PBS for 30 min at 37 °C and 5% CO_2_. As a negative control, we used cells on TCP incubated with dimethyl sulfoxide for 20 min. Scaffolds were imaged using an IX 51 inverted fluorescence microscope (Olympus, Japan). Four images were captured per film. DNA was quantified at each time point after overnight digestion with 50 μg/mL proteinase K in 100 mM K_2_HPO_4_ at pH 8.0 and at 56 °C. After proteinase K deactivation through heating (at 90 °C for 10 min), the samples were centrifuged and the supernatants were analyzed with the Quant-iT PicoGreen dsDNA assay kit (Invitrogen, Thermo Fisher Scientific, Ireland), as per manufacturer’s protocol. A standard curve was used to infer the DNA concentration in the samples.

The collagen scaffolds were cut in circular shapes, placed in 24-well plates, sterilized as described above, and incubated with RPMI-1640 media supplemented with 1% penicillin/streptomycin and 10% fetal bovine serum (RPMI complete media) for 3 days. Media incubated with the scaffolds (preconditioned media) were collected and filtered through 0.2 μm sterile filters. Human-derived leukemic monocyte cells (THP-1, American Type Culture Collection, United Kingdom) were seeded on TCP and on the scaffolds at a density of 26 × 10^3^ cell/cm^2^ in RPMI complete media supplemented with 100 ng/mL of phorbol 12-myristate 13-acetate to induce an adherent mature macrophage-like state for 6 h, as has been described previously.^34–36^ Nonadherent cells were washed off with 1× PBS. Adherent cells on TCP were incubated with the filtered preconditioned media, while adherent cells on the scaffolds were incubated with RPMI complete media. Lipopolysaccharide (LPS) from *Escherichia coli* O55:B5 was utilized at 100 ng/mL as a positive control for macrophage activation on TCP. Macrophages seeded on TCP and cultured with RPMI complete media were used as a negative control. Cells were cultured at 37 °C and 5% CO_2_ for 24 and 48 h. At each time point, THP-1 metabolic activity and DNA concertation were assessed as described above. Cells on the scaffolds were fixed with 10% formalin, permeabilized with 0.2% Triton X, and stained with rhodamine-conjugated phalloidin (Thermo Fisher Scientific, Ireland) and 4′6′-diamino-2-phenylindole. Cells on the scaffolds were imaged with an IX 51 inverted fluorescence microscope (Olympus, Japan). Macrophage activation was inferred by cell elongation analysis using ImageJ (National Institutes of Health, United States of America). Briefly, the perimeter of 25–50 cells per image (five images per sample) was manually circled to obtain the aspect ratio, circularity, and roundness values. Macrophages with an aspect ratio higher than 3.0 or with an aspect ratio higher than 3.0 and circumference and roundness lower than 0.5 were considered elongated. Results are displayed as % of elongated cells. TNF-α inflammatory cytokine in the supernatant was measured using a DY210 DuoSet ELISA (Bio-Techne, United States of America) assay, as per the manufacturer’s guidelines. Cytokine concentration was normalized to DNA concentration for each sample.

### 2.7 Statistical Analysis

Numerical data are expressed as mean ± standard deviation. Data were analyzed using the Prism v10.1.1 (GraphPad Software Inc., United States of America) software. Student’s *t*-test and analysis of variance (ANOVA) followed by Fisher’s posthoc test were performed for sample populations showing normal distribution (Kolmogorov–Smirnov normality test) and equality of variances (Levine’s test for homogeneity of variance). Nonparametric Mann–Whitney *U* and Kruskal–Wallis tests were used when the populations were not normally distributed and/or were not of equal variance. Statistical significance was accepted at *p* <0.05.

## 3 Results

### 3.1 Evaluation of Collagen Preparation Purity and Chemical Properties

Electrophoresis analysis (Figure 1A) revealed that all collagen preparations exhibited a typical collagen type I electrophoretic pattern, characterized by α1 and α2 chains in its monomeric (α-bands), dimeric (β-bands), and trimeric (γ-bands) forms, with no major differences in purity as a function of tissue origin. FTIR analysis (Figure 1B) made apparent that all collagen preparations had typical collagen type I spectra with well-defined peaks at ∼3300 cm^–1^ (amide A), ∼1631 cm^–1^ (amide I), ∼1547 cm^–1^ (amide II), and ∼1236 cm^–1^ (amide III). A peak at ∼2924 cm^–1^, related to the CH_2_ asymmetrical stretching of amide B, was only evidenced in the caprine collagen samples. Helix integrity, determined by the absorbance ratio between the peak heights of amide III and ∼1450 cm^–1^, was in the 0.97 ± 0.05 (Table S1) range for all the samples, indicating that none of the collagen preparations had been denatured.

**Figure 1:**
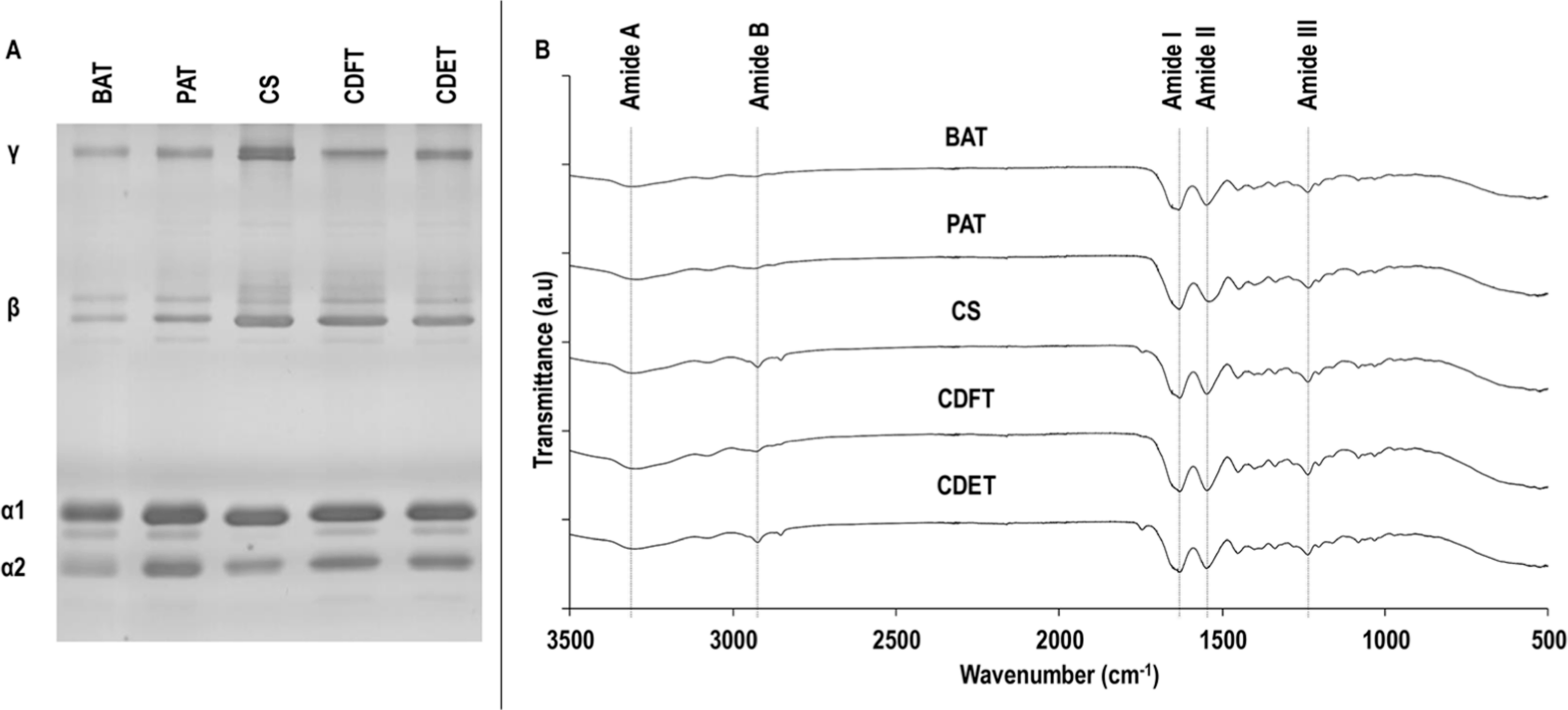
Electrophoretic mobility (A) and FTIR spectra (B) of collagen type I isolated and purified from bovine Achilles tendon (BAT), porcine Achilles tendon (PAT), caprine skin (CS), caprine digital flexor tendon (CDFT), and caprine digital extensor tendon (CDET). *N* = 1.

### 3.2 Evaluation of Collagen Film Physicochemical Properties

Macroscopic analysis revealed that all films, independently of the collagen origin, were transparent when not cross-linked and slightly opaquer after cross-linking (Figure 2A). AFM analysis revealed a fibrous surface topography for the non-cross-linked films and a slightly smoother surface topography after cross-linking, both for all collagen preparations (Figure 2B). Quantitative fibril diameter analysis revealed that the bovine and porcine preparations yielded the thinnest (*p* <0.05) and thickest (*p* <0.05), respectively, fibrils; within the caprine groups, no significant (*p* >0.05) differences in the collagen fibril diameter were observed; and, in all cases, cross-linking significantly (*p* <0.05) decreased the fibril diameter (Table 1). With respect to surface roughness, among the non-cross-linked groups, the BAT and the CDET preparations resulted in the lowest (*p* <0.05) and highest (*p* <0.05), respectively, surface roughness; cross-linking significantly (*p* <0.05) reduced surface roughness for the PAT, the CDFT, and the CDET preparations; and within the caprine groups, no significant (*p* >0.05) differences in surface roughness were observed between the tendon preparations in the non-cross-linked and cross-linked state and the skin preparation resulted in significantly (*p* <0.05) higher surface roughness than the tendon preparations in the cross-linked state (Table 1). Free amine analysis revealed that among the non-cross-linked groups, the BAT and the CDET preparations exhibited the highest (*p* <0.05) and lowest (*p* <0.05), respectively, free amine content and cross-linking significantly (*p* <0.05) decreased free-amine content in all scaffolds (Table 1). Thickness analysis made apparent that the BAT preparation induced the thickest (*p* <0.05) scaffolds in both non-cross-linked and cross-linked state; and cross-linking only significantly (*p* <0.05) increased the thickness of the CS scaffolds (Table 1). Tensile testing analysis of the non-cross-linked scaffolds revealed no significant (*p* >0.05) differences in stress at break between the groups and the CS, the CDFT, and the CDET scaffolds had significantly (*p* <0.05) lower and higher strain at break and Young’s modulus, respectively, values than the BAT and the PAT scaffolds (Table 1). Cross-linking significantly (*p* <0.05) increased stress at break of all scaffolds, strain at break of all caprine scaffolds, and Young’s modulus of the BAT and PAT scaffolds (Table 1).

**Figure 2:**
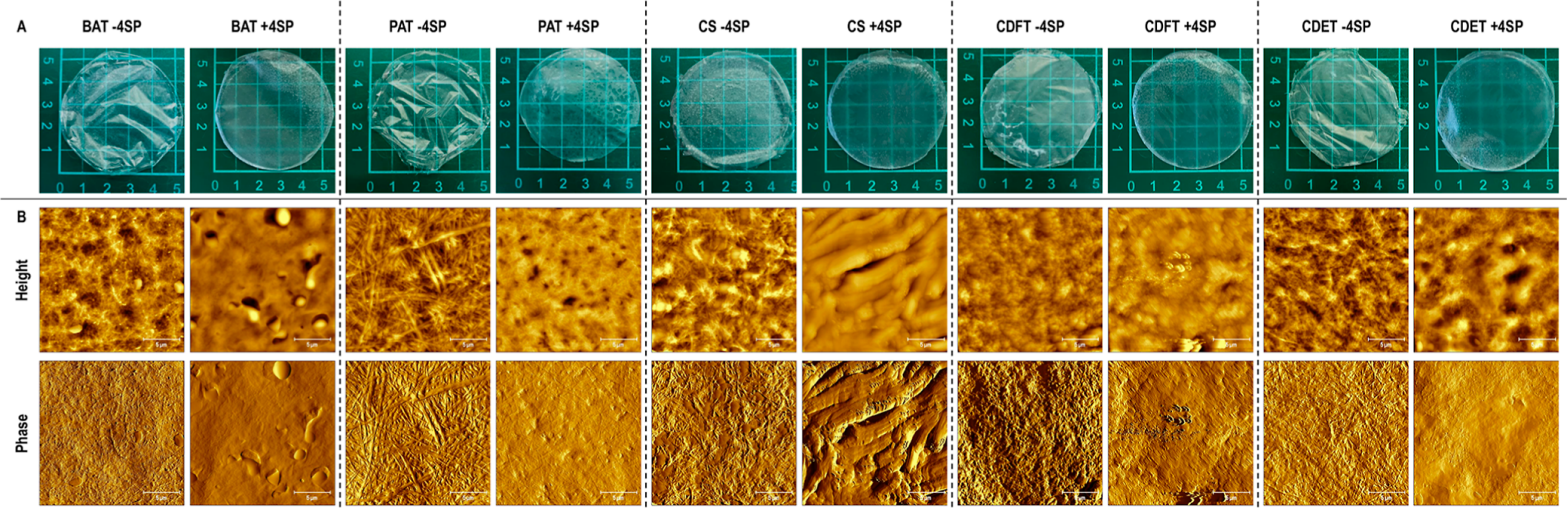
Macroscopic (A) and AFM (B) images of non-cross-linked (−4SP) and cross-linked (+4SP) bovine Achilles tendon (BAT), porcine Achilles tendon (PAT), caprine skin (CS), caprine digital flexor tendon (CDFT), and caprine digital extensor tendon (CDET) collagen scaffolds. Macroscopic image scale bar: 5 cm. AFM image scale bar: 5 μm. *N* = 3.

**Table 1:** Collagen Fibril Diameter, Surface Roughness, Free Amines, Thickness, Stress at Break, Strain at Break, and Young’s Modulus of Non-crosslinked (−4SP) and Crosslinked (+4SP) bovine Achilles Tendon (BAT), Porcine Achilles Tendon (PAT), Caprine Skin (CS), Caprine Digital Flexor Tendon (CDFT), and Caprine Digital Extensor Tendon (CDET) Collagen Scaffolds^a^

	collagen fibril diameter (nm)	surface roughness (nm)	free amines (ng/mg)	thickness (mm)	stress at break (kPa)	strain at break (%)	Young’s modulus (MPa)
BAT −4SP	104 ± 25	45.8 ± 4.6	3.65 ± 0.52^#^	0.11 ± 0.02*	154 ± 41	5.17 ± 1.99*	3.75 ± 1.26
BAT +4SP	53 ± 13^&^	35.5 ± 6.7	0.68 ± 0.06^&^	0.17 ± 0.06^#^	1392 ± 492^&^	4.10 ± 0.57	55.01 ± 15.68^&^
PAT −4SP	337 ± 121^#^	65.9 ± 12.7	3.06 ± 0.36	0.09 ± 0.02	116 ± 72	8.51 ± 4.56*	1.72 ± 0.90
PAT +4SP	160 ± 54^&^	30.5 ± 3.7^&^	0.93 ± 0.12^&^	0.09 ± 0.01	815 ± 133^&^	7.26 ± 1.62	18.80 ± 4.62^&^
CS −4SP	258 ± 71	66.9 ± 6.3	2.51 ± 0.32	0.05 ± 0.02	153 ± 120	0.52 ± 0.19	20.14 ± 11.17*
CS +4SP	145 ± 39^&^	76.7 ± 35.9*	0.70 ± 0.07^&^	0.09 ± 0.01^&^	446 ± 136^&^	4.47 ± 1.74^&^	17.42 ± 5.48
CDFT −4SP	183 ± 49	73.7 ± 12.6	2.34 ± 0.12	0.05 ± 0.03	91 ± 42	0.84 ± 0.16	11.96 ± 4.71*
CDFT +4SP	137 ± 55^&^	35.8 ± 4.5^&,^*	0.78 ± 0.19^&^	0.08 ± 0.01	585 ± 256^&^	5.42 ± 1.88^&^	17.55 ± 4.88
CDET −4SP	226 ± 67	87.4 ± 8.4^#^	2.24 ± 0.55	0.05 ± 0.02	248 ± 123	1.22 ± 0.19	25.68 ± 12.87*
CDET +4SP	125 ± 37^&^	44.7 ± 10.1^&^,*	0.65 ± 0.11^&^	0.08 ± 0.01	511 ± 262^&^	3.80 ± 1.47^&^	21.45 ± 8.21

a # indicates highest (*p* <0.05) population. * indicates significantly (*p* <0.05) higher populations. & indicates significantly (*p* <0.05) lower/higher than crosslinked. *N* = 5.

Across all collagen preparations, resistance to enzymatic digestion revealed, in general, a % weight reduction across all scaffolds as a function of exposure time to collagenase (at 48 h, all groups exhibited significantly, *p* <0.05, lower % weight to respective groups at 0 h); cross-linking to increase resistance to collagenase digestions (at 48 h, all cross-linked groups exhibited significantly, *p* <0.05, higher % weight to respective non-cross-linked groups); no significant (*p* >0.05) differences as a function of collagen origin within the non-cross-linking state at a given time point; and among the cross-linked scaffolds, the CS scaffolds exhibited a significantly (*p* <0.05) lower % of remaining weight compared to the other cross-linked groups at 24 h, 36 h, and 48 h (Figure 3).

**Figure 3:**
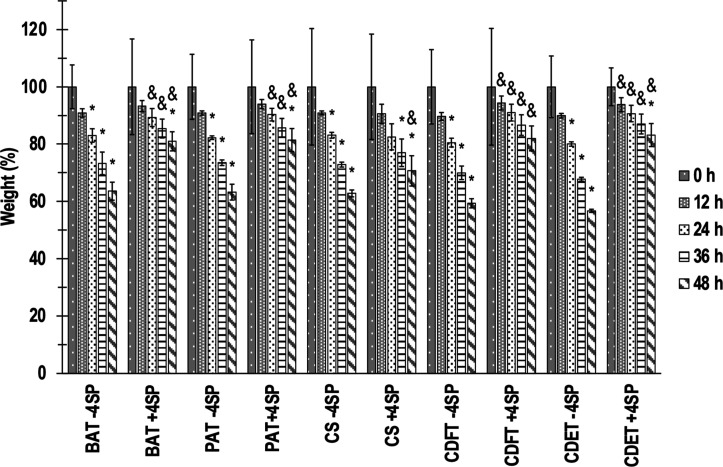
Weight (%) loses of non-cross-linked (−4SP) and cross-linked (+4SP) bovine Achilles tendon (BAT), porcine Achilles tendon (PAT), caprine skin (CS), caprine digital flexor tendon (CDFT), and caprine digital extensor tendon (CDET) collagen scaffolds as a function of exposure time (0 h, 12 h, 24 h, 36 h, and 48 h) to collagenase. * indicates significantly (*p* <0.05) lower to 0 h. & indicates significantly (*p* <0.05) higher than the corresponding non-cross-linked group. # indicates significantly (*p* <0.05) lower than other cross-linked groups. *N* = 3.

### 3.3 Evaluation of Collagen Film Biological Properties Using Human Skin Fibroblasts and Human Macrophages

Qualitative human skin fibroblast viability analysis revealed that all scaffolds induced similar cell viability at all time points, and at day 7, the whole area was covered by the cells (Figure S1). DNA quantification revealed that among the non-cross-linked groups, the CS preparation induced the highest (*p* <0.05) DNA content at all time points and among the cross-linked groups, the PAT, the CDET, and the CDFT induced significantly (*p* <0.05) higher DNA content than the other preparations only at day 7 (Figure 4A). Cell metabolic activity analysis revealed no significant (*p* >0.05) differences between the non-cross-linked groups at day 3 and day 5 and at day 7, the BAT preparation induced the highest (*p* <0.05) cell metabolic activity and no significant (*p* >0.05) differences between the cross-linked groups at any time point (Figure 4B).

**Figure 4:**
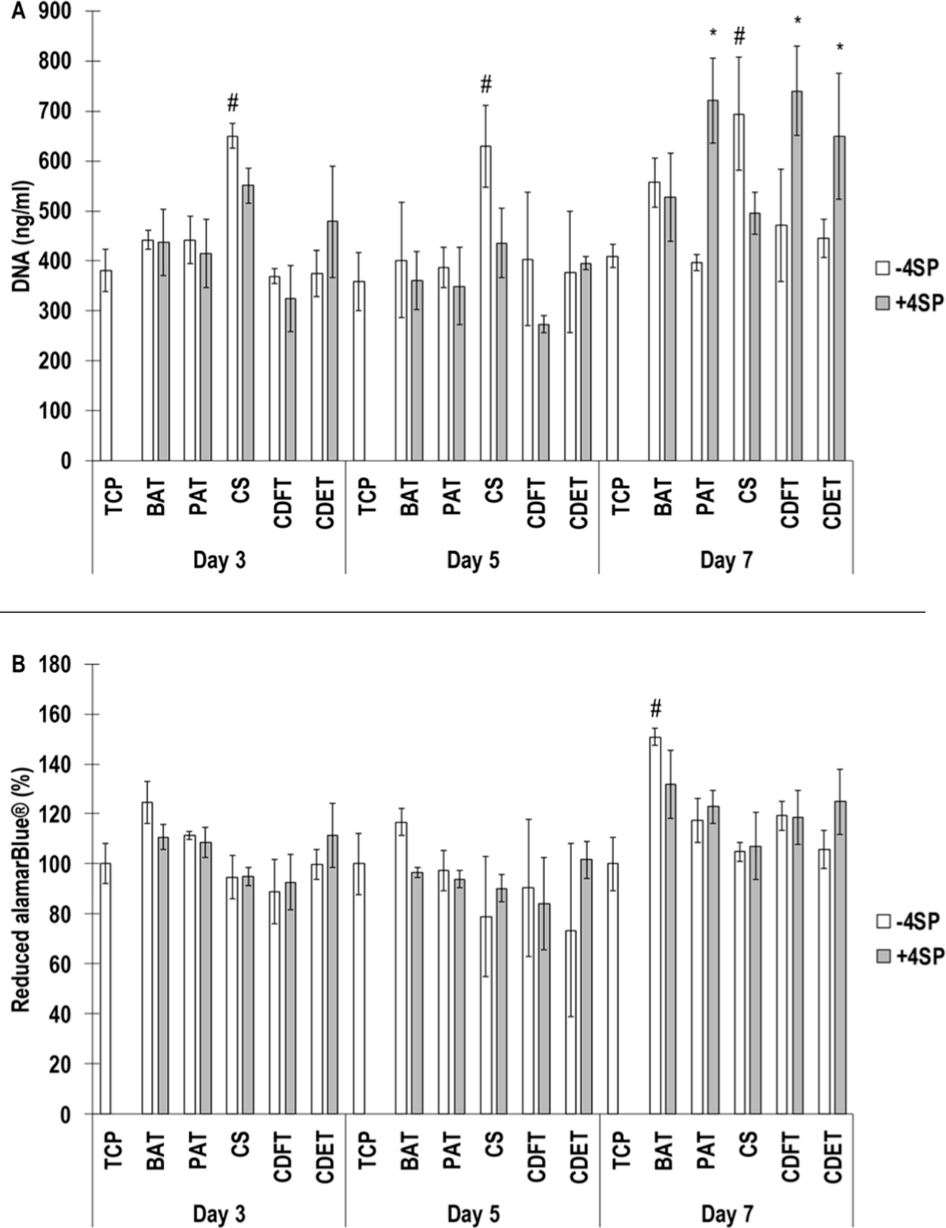
DNA (A) and reduced alamarBlue of WS1 fibroblasts cultured for 3, 5, and 7 days on non-cross-linked (−4SP) and cross-linked (+4SP) bovine Achilles tendon (BAT), porcine Achilles tendon (PAT), caprine skin (CS), caprine digital flexor tendon (CDFT), and caprine digital extensor tendon (CDET) collagen scaffolds. # indicates highest (*p* <0.05) population. * indicates significantly (*p* <0.05) higher populations. *N* = 3.

Direct cultures with human macrophages revealed that the CS preparation induced the highest (*p* <0.05) DNA content at both time points in both non-cross-linked and cross-linked states and cross-linking significantly (*p* <0.05) reduced DNA content of the CS and CDET preparations at day 1 and of the PAT, the CS, the CDFT, and the CDET preparations at day 2 (Figure 5A). Metabolic activity analysis showed no significant (*p* >0.05) differences as a function of collagen preparation and cross-linking state at any time point and, in general, the TCP and LPS groups induced significantly (*p* <0.05) higher metabolic activity than the other groups at both time points (Figure 5B). Qualitative (Figure S2) and quantitative (Figure 5C) macrophage morphology analyses revealed that all conditions had less than 5% elongated cells at day 1 and LPS induced the highest (*p* <0.05) % of elongated cells at day 2, with no significant (*p* >0.05) differences between the collagen groups as a function of collagen preparation and cross-linking state. TNF-α analysis revealed that LPS induced the highest (*p* <0.05) TNF-α release at day 1 and no significant (*p* >0.05) differences were observed between the collagen groups as a function of collagen preparation and cross-linking state and at day 2, LPS and cross-linked PAT and cross-linked CS induced significantly (*p* <0.05) higher TNF-α release than the other groups (Figure 5D).

**Figure 5:**
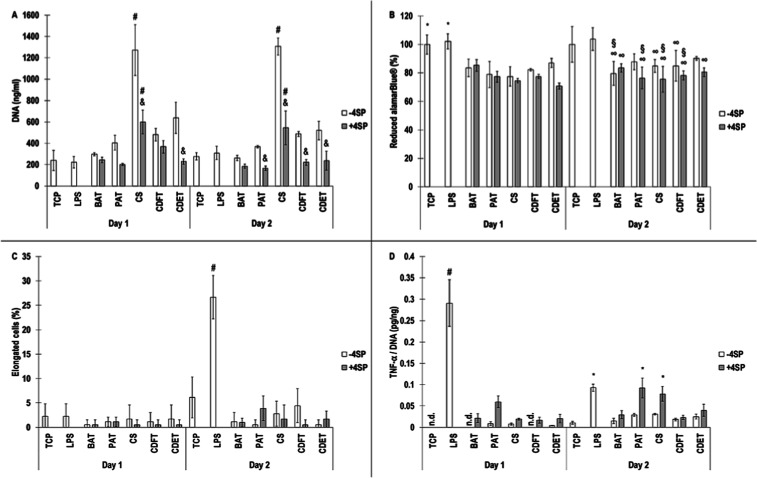
DNA (A), reduced alamarBlue, elongated cells, and TNF-α/DNA of direct human macrophage cultures for 1 and 2 days on tissue culture plastic (TCP), on lipopolysaccharide (LPS)-treated TCP, and on non-cross-linked (−4SP) and cross-linked (+4SP) bovine Achilles tendon (BAT), porcine Achilles tendon (PAT), caprine skin (CS), caprine digital flexor tendon (CDFT), and caprine digital extensor tendon (CDET) collagen scaffolds. # indicates highest (*p* <0.05) population. * indicates significantly (*p* <0.05) higher populations. & indicates significantly (*p* <0.05) lower than not cross-linked. § indicates significantly (*p* <0.05) lower than TCP. ∞ indicates significantly (*p* <0.05) lower than LPS. n.d. indicates not detected. *N* = 3.

Indirect cultures with human macrophages revealed no significant (*p* >0.05) differences in DNA content between the groups at a given time point (Figure S3A). In general, at both time points, LPS induced significantly (*p* < 0.05) higher metabolic activity than the other groups and no significant (*p* >0.05) differences were observed as a function of collagen preparation and cross-linking state (Figure S3B). With respect to cell elongation, qualitative (Figure S4) and quantitative (Figure S3C) analyses revealed no significant (*p* >0.05) differences between the groups at day 1 and at day 2, LPS induced the highest (*p* <0.05) % of elongated cells and no significant (*p* >0.05) differences were observed as a function of collagen preparation and cross-linking state. TNF-α analysis revealed that LPS induced the highest (*p* <0.05) TNF-α release at day 1 and day 2 and no significant (*p* >0.05) differences were observed between the collagen groups as a function of collagen preparation and cross-linking state (Figure S3D).

## 4 Discussion

Collagen type I is a building block for many medical devices. Nonhuman animal tissues remain the primary source of collagen type I. Unfortunately, the two principal species utilized for this endeavor (i.e., bovine and porcine) entail drawbacks associated with potential interspecies disease transmission, religious constraints, and their breeding involves high antibiotic usage that contributes to antibiotic resistance in humans. In this context, caprine tissues may represent a viable alternative source for collagen type I due to its worldwide availability and its lower associated risk of zoonosis. Herein, collagen type I was isolated from PAT, BAT, CS, CDFT, and CDET and the purity of the preparations and the physicochemical and biological properties of the derived scaffolds (non-cross-linked and cross-linked) were assessed.

### 4.1 Evaluation of Collagen Preparation Purity and Chemical Properties

Electrophoresis analysis confirmed the presence of collagen type I in all samples, as evidenced by the characteristic α1 and α2 bands.^29^ Consistent with the heterotrimeric structure of collagen type I, comprising two α1 and one α2 chain,^37^ the α1 band was denser than the α2 band across all samples. Higher molecular weight β and γ bands, corresponding to cross-linked α chain dimmers and trimmers,^38^ respectively, were also observed. These bands were more pronounced in caprine-derived samples, regardless of the tissue of origin, suggesting a greater presence of intramolecular cross-links,^39,40^ possibly reflecting species-specific or age-related differences in collagen maturation.^18,41^ However, this interpretation remains limited by the qualitative nature of SDS-PAGE. The electrophoretic conditions employed, 3% stacking and 5% resolving acrylamide gels, proved effective in resolving the primary collagen type I components. These gel concentrations have been previously validated for collagen analysis from bovine,^18,42,43^ porcine,^18,40,44^ piscine,^45^ and in vitro-derived sources.^46–49^ For finer resolution of lower molecular fragments or degradation products, resolving gels of higher acrylamide percentage (e.g., 8%,^50–53^ 10%,^51,54^ or 12% ^38,55^ ) may be more appropriate. FTIR analysis revealed that all the extracts, irrespective of their tissue or species of origin, exhibited the characteristic spectral features of native collagen type I.^56^ Prominent peaks were observed at ∼3300 cm^–1^, ∼1631 cm^–1^, ∼1547 cm^–1^, and ∼1236 cm^–1^, corresponding to amide A (N–H stretching), amide I (C=O stretching), amide II (N–H bending coupled with C–N stretching), and amide III (C–N stretching and N–H bending, with contributions from glycine and proline CH_2_ wagging vibrations), respectively.^57^ The absence of significant shifts or changes in peak intensities among the samples suggests that the functional groups and the backbone structure remained largely conserved across collagen extracts.^58,59^ A minor peak at ∼2924 cm^–1^, likely associated with CH_2_ asymmetrical stretching (amide B), was only appreciable in the caprine collagen samples. This may result from divergences in sample humidity at the time of testing, which have been shown to influence absorbance at this wavelength.^60^ The amide III to 1450 cm^–1^ intensity ratio, a sensitive indicator of triple helix integrity,^61,62^ remained close to 1.0 and consistent across samples (0.97 ± 0.05), indicating the extraction protocol preserved the native triple helical conformation.^63^ Overall, these findings indicate that the acid- and pepsin-based extraction methods yielded high-purity collagen type I, regardless of the source tissue or species.

### 4.2 Evaluation of Collagen Film Physicochemical Properties

Starting with macroscopic appearance, no differences were observed as a function of collagen origin and cross-linking appeared to result in a smooth scaffold surface, which is in agreement with previous publications that have shown a cross-linking agent-dependent surface morphology.^32,64^ Fibril diameter analysis revealed that the PAT preparation resulted in scaffolds with the thickest fibrils, the caprine preparations resulted in scaffolds with an intermediate fibril diameter, and the BAT preparation resulted in scaffolds with the thinnest fibrils. As within the caprine tissues (skin and tendons) no differences were observed in the fibril diameter, it is safe to assume that the observed differences are due to species-dependent differences in collagen self-assembly dynamics, in accordance with previous reports.^23^ Across all collagen preparations, cross-linking decreased the fibril diameter and in general surface roughness (mirroring macroscopic observation), as has been reported previously and was attributed to the formation of intermolecular cross-links, the breaking up of hydrogen bonds, and the removal of water.^43,65^ Surface roughness is a key parameter in scaffold design as it can significantly influence biological responses, including adhesion, morphology, and stem cell differentiation by altering integrin engagement and focal adhesion dynamics.^66–68^

With respect to free amines, in the non-cross-linked state, no significant differences were observed between the caprine tissues and the caprine tissues had lower free amines than their bovine and porcine counterparts. Cross-linking reduced free amine content across all preparations, as has been well established in the literature with 4SP^27,69^ and other cross-linking agents.^70,71^ Scaffold thickness was highest in BAT films despite their thinner fibrils, possibly due to higher solution viscosity reducing solvent evaporation during film formation.^72,73^ Collagen cross-linking with 4SP caused a general trend toward increased thickness, significantly only in CS scaffolds. This may be attributed to hysteric hindrance introduced by the branched cross-linker^74^ and enhanced water retention from its hydrophilic structure,^75^ both of which may reduce fibril compaction and maintain a more expanded matrix. Mechanical analysis of the non-cross-linked scaffolds revealed the BAT scaffolds had higher (albeit not significant) stress at break values than the PAT scaffolds, the caprine scaffolds exhibited significantly lower strain at break values and significantly higher Young’s modulus values than their porcine and bovine scaffolds, and within the caprine tissues, the CDET scaffolds had higher (albeit not significant for all) stress at break, strain at break, and Young’s modulus values than the CS and CDFT. In general, all these observations are in accordance with previous publications. For example, a previous report^18^ has shown porcine collagen sponges to have lower stress and modulus values than bovine collagen sponges and tendon-derived collagen scaffolds to have higher stress and modulus values than skin-derived collagen scaffolds and the authors attributed these differences to age- and exercise-related cross-linking^76,77^ and on the different packing of collagen fibrils and fibers in different tissues.^78–81^ Another study argued that tissue function is an important contributor to the mechanical properties of collagen scaffolds, as reconstituted collagen fibers fabricated from BAT collagen had higher force and strain at break than reconstituted collagen fibers fabricated from rat tail tendon collagen.^23^ Authors have also argued that the thickness of a given tissue is responsible for its stiffness, considering that human corneal tissues are stiffer than porcine corneal tissues as the former are thinner than the latter.^82^ One should also note that another study has reported differences in the mechanical properties of equine DFTs and DETs as a function of breed and within the same breed, the DFTs had higher load at break and strain at break values and lower stress at break and modulus values than the DETs,^83^ again showing function-dependent mechanical properties. Similarly, differences in elastic modulus and collagen fibril radius of Achilles tendon from two different inbred mouse strains have been shown.^84^ Of course, pathologies are also responsible for the mechanical properties of a tissue, considering that previous studies have shown healthy tendons to have lower modulus and higher transition strain than insertional Achilles tendinopathy tendons.^85^ Taken together, the mechanical properties of tissues depend, among others, on species, breed, tissue, and health state. The inherently high stiffness of caprine scaffolds, relative to their bovine and porcine counterparts, positions them as promising candidates for applications demanding mechanically robust matrices, potentially eliminating the need for additional cross-linking agents that may be costly or cytotoxic.^86^

Cross-linking significantly increased stress at break of all scaffolds, as it has been well documented in the literature for a wide range of collagen scaffolds (e.g., sponges,^45,87,88^ films,^89–91^ hydrogels).^92,93^ With respect to strain at break and Young’s modulus values, a clear trend across all scaffolds was not observed. Specifically, 4SP cross-linking significantly increased the strain at break in caprine scaffolds without altering their Young’s modulus, regardless of the tissue origin, whereas bovine and porcine scaffolds exhibited an opposite trend, with increased stiffness but no improvement in extensibility. Collectively, these findings suggest that caprine collagen may possess a higher baseline level of mature collagen cross-links than bovine and porcine collagen, as evidenced by the thicker polymeric bands in SDS-PAGE,^39,40,94^ lower free amine content,^20,40^ and higher scaffold stiffness^95^ prior to cross-linking. The attenuated efficacy of 4SP cross-linking in stiffening caprine scaffolds may thus reflect the reduced availability of reactive amines due to the presence of pre-existing bonds. Conversely, the branched and hydrophilic nature of 4SP may have promoted increased matrix hydration and interfibrillar spacing, potentially accounting for the enhanced extensibility observed in caprine scaffolds.^75^ In contrast, the greater abundance of reactive free amines in bovine and porcine scaffolds likely facilitated more extensive 4SP-mediated cross-linking, resulting in a stiffer, less compliant matrix.^96,97^ Validation of this hypothesis will require direct quantification of mature cross-link species, such as pyridinoline and deoxypyridinoline, via high-performance liquid chromatography and mass spectrometry.^98^ The outcome also aligns with previous studies showing variable effects of collagen cross-linking depending on the cross-linker agent utilized. For example, glutaraldehyde, genipin, and oleuropein decreased strain at break and increased elastic modulus; carbodiimide increased strain at break and decreased elastic modulus; and 4SP decreased strain at break and elastic modulus of collagen films.^43^ For reconstituted collagen fibers, glutaraldehyde and genipin increased strain at break and modulus; carbodiimide increased strain at break and decreased modulus; and epoxide decreased strain at break and increased modulus.^65^ For collagen sponges, genipin and diphenyl phosphoryl azide were shown to increase modulus and to decrease strain at break, while carbodiimide was shown to increase modulus and to not affect strain at break.^99^

Collagenase degradation profiles revealed no significant differences among non-cross-linked scaffolds, challenging the hypothesis of higher native cross-link density in caprine collagen, which would be expected to enhance resistance to enzymatic cleavage.^44^ While 4SP cross-linking significantly improved degradation resistance at 48 h across samples, cross-linked CS scaffolds exhibited faster degradation at earlier time points. This may reflect the limited efficacy of 4SP in CS scaffolds, potentially due to their reduced free-amine availability, as previously discussed.^20,40^

### 4.3 Evaluation of Collagen Film Biological Properties Using Human Skin Fibroblasts and Human Macrophages

Although biological analysis with human skin fibroblasts (direct culture) and human macrophages (direct and indirect cultures) revealed some differences, we do not consider them to be of importance as they were not consistent among tissues from which the collagen preparations were derived from, cross-linking state, cell type, and time point. In general, all produced scaffolds supported human skin fibroblast growth, as it would have expected, considering that the 4SP has been extensively used to stabilize collagen scaffolds without any in vitro^27,100^ or in vivo^101,102^ side effects. With respect to human macrophage response, all collagen preparations were produced using the acid/pepsin extraction protocol that is known to induce low macrophage response.^22^ Some differences in TNF-α were observed between non-cross-linked and cross-linked groups (i.e., PAT and CS); we attribute this to the increased surface rigidity (a known modulator of macrophage polarization^103,104^ ) due to the cross-linking and not to a direct effect of the 4SP, as this was not observed in the indirect cultures.

## 5 Conclusions

In the quest for alternative to porcine and bovine collagen type I sources for medical device development, herein we venture to assess the potential of caprine collagen type I. Based on purity analysis and chemical characterization of collagen type I preparations and physiochemical and biological properties of derived scaffolds, we conclude that caprine collagen type I preparations from skin, digital flexor tendon, and digital extensor tendon and respective scaffolds have proportional characteristics to porcine Achilles tendon and bovine Achilles tendon preparations and scaffolds. Our findings clearly illustrate the potential of caprine tissues for the extraction of collagen type I and subsequent deployment in medical device development.

## Supplementary Material



## Data Availability

Raw and processed data are available upon request from Ignacio Sallent.

## Notes

Triple helix integrity ratio for the different collagen preparations as determined from FTIR spectra; live and dead cell images of human WS1 fibroblasts; rhodamine-conjugated phalloidin and 4′6′-diamino-2-phenylindole stained human macrophages (direct cultures); DNA, alamarBlue, elongated cells, and TNF-α/DNA analyses of human macrophages (indirect cultures); and rhodamine-conjugated phalloidin and 4′6′-diamino-2-phenylindole stained human macrophages (indirect cultures) (PDF)
