# Supplementary material for: Assessing the Potential of Caprine Collagen Type I in the Development of Medical Devices
Source: Biomacromolecules. 2025 Sep 1;26(10):6418–29. doi: 10.1021/acs.biomac.5c00309 (PMC12522132; doi:10.1021/acs.biomac.5c00309)

Supplementary Information

Title

Assessing the potential of caprine collagen type I in the development of medical devices

Authors

Ignacio Sallent (1), Arely Leon Lopez (2), Gabriel Aguirre-Álvarez (3), Dimitrios I Zeugolis* (1, 4)

Affiliations

(1) Regenerative, Modular & Developmental Engineering Laboratory (REMODEL) and CÚRAM Research Ireland Centre for Medical Devices, University of Galway, Galway, H91 TK33, Ireland

(2) TecNM Campus Venustiano Carranza, Puebla, 73049, Mexico

(3) Agricultural Sciences Institute, Autonomous University of Hidalgo State, Hidalgo, 43775, Mexico

(4) Regenerative, Modular & Developmental Engineering Laboratory (REMODEL), Charles Institute of Dermatology, Conway Institute of Biomolecular & Biomedical Research and School of Mechanical & Materials Engineering, University College Dublin (UCD), Dublin, D04 V1W8, Ireland

*Corresponding Author: Dimitrios I Zeugolis, REMODEL, UCD, Dublin, D04 V1W8, Ireland. Tel: +353 17 16 18 87. Email: dimitrios.zevgolis@ucd.ie

Table S1: Triple helix integrity ratio for the different collagen preparations, as determined from FTIR spectra. N = 1.

Collagen preparation	Triple helix integrity ratio
BAT	0.97
PAT	1.02
CS	0.93
CDFT	0.95
CDET	0.98
Overall	0.97 ± 0.03

Figure S1: Live (green) and dead (red) images of WS1 fibroblasts cultured for 3, 5 and 7 days on non-crosslinked (-4SP) and crosslinked (+4SP) bovine Achilles tendon (BAT), porcine Achilles tendon (PAT), caprine skin (CS), caprine digital flexor tendon (CDFT) and caprine digital extensor tendon (CDET) collagen scaffolds. Scale bar: 100 µm. N = 3.

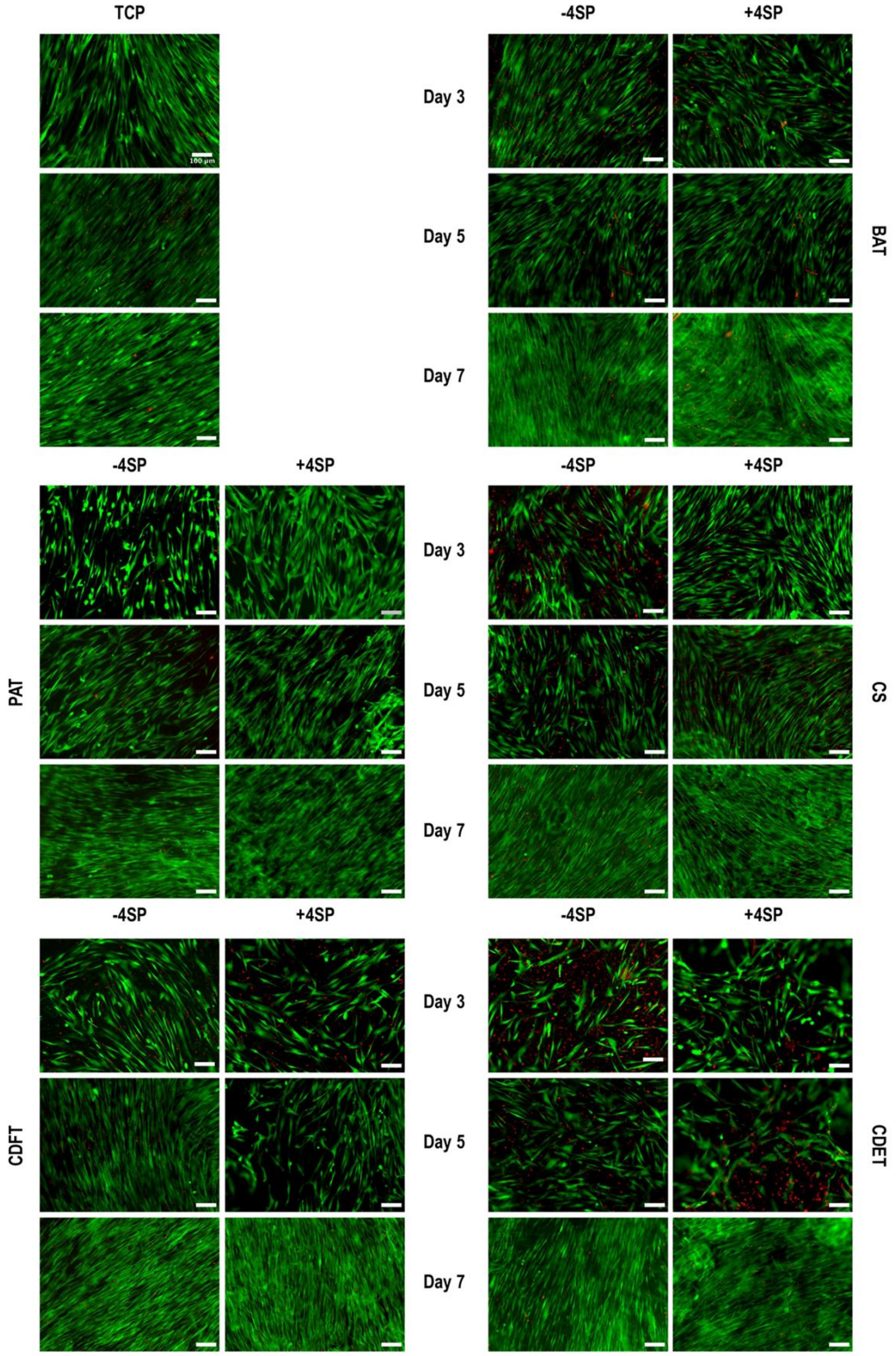

Figure S2: Rhodamine-conjugated phalloidin (red) stained cytoskeleton and 4' 6'-diamino-2-phenylindole (blue) stained nuclei of direct human macrophage cultures for 1 and 2 days on tissue culture plastic (TCP), on lipopolysaccharide (LPS) treated TCP and on non-crosslinked (-4SP) and crosslinked (+4SP) bovine Achilles tendon (BAT), porcine Achilles tendon (PAT), caprine skin (CS), caprine digital flexor tendon (CDFT) and caprine digital extensor tendon (CDET) collagen scaffolds. #: indicates highest ($p < 0.05$) population. White arrows indicate elongated cells. N = 3.

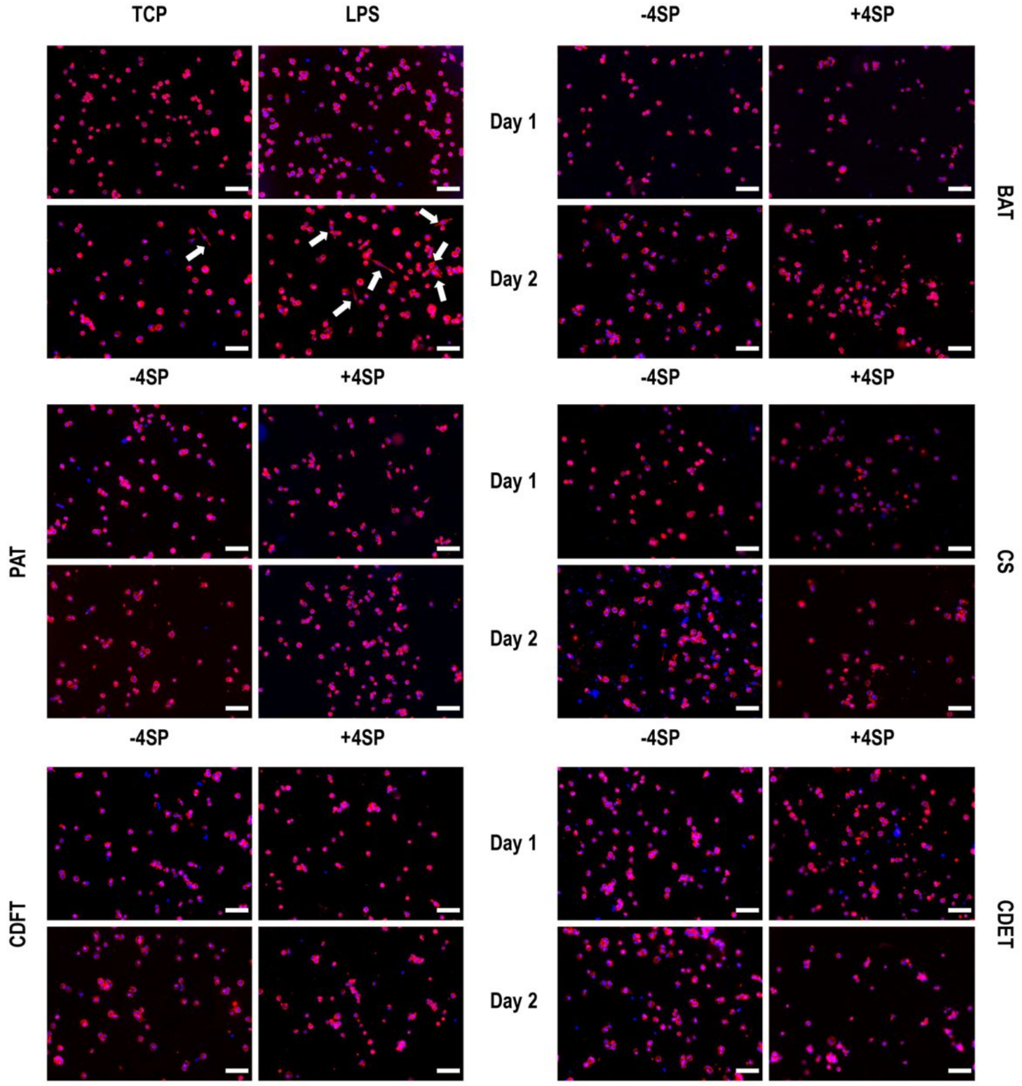

Figure S3: DNA (A), reduced alamarBlue®, elongated cells and TNF-α / DNA of indirect human macrophage cultures for 1 and 2 days on tissue culture plastic (TCP), on lipopolysaccharide (LPS) treated TCP and on non-crosslinked (-4SP) and crosslinked (+4SP) bovine Achilles tendon (BAT), porcine Achilles tendon (PAT), caprine skin (CS), caprine digital flexor tendon (CDFT) and caprine digital extensor tendon (CDET) collagen scaffolds. #: indicates highest ($p < 0.05$) population. *: indicates significantly ($p < 0.05$) higher populations. &: indicates significantly ($p < 0.05$) lower to not crosslinked. §: indicates significantly ($p < 0.05$) lower to TCP. ∞: indicates significantly ($p <$ 0.05) lower to LPS. n.d.: indicates not detected. N = 3.

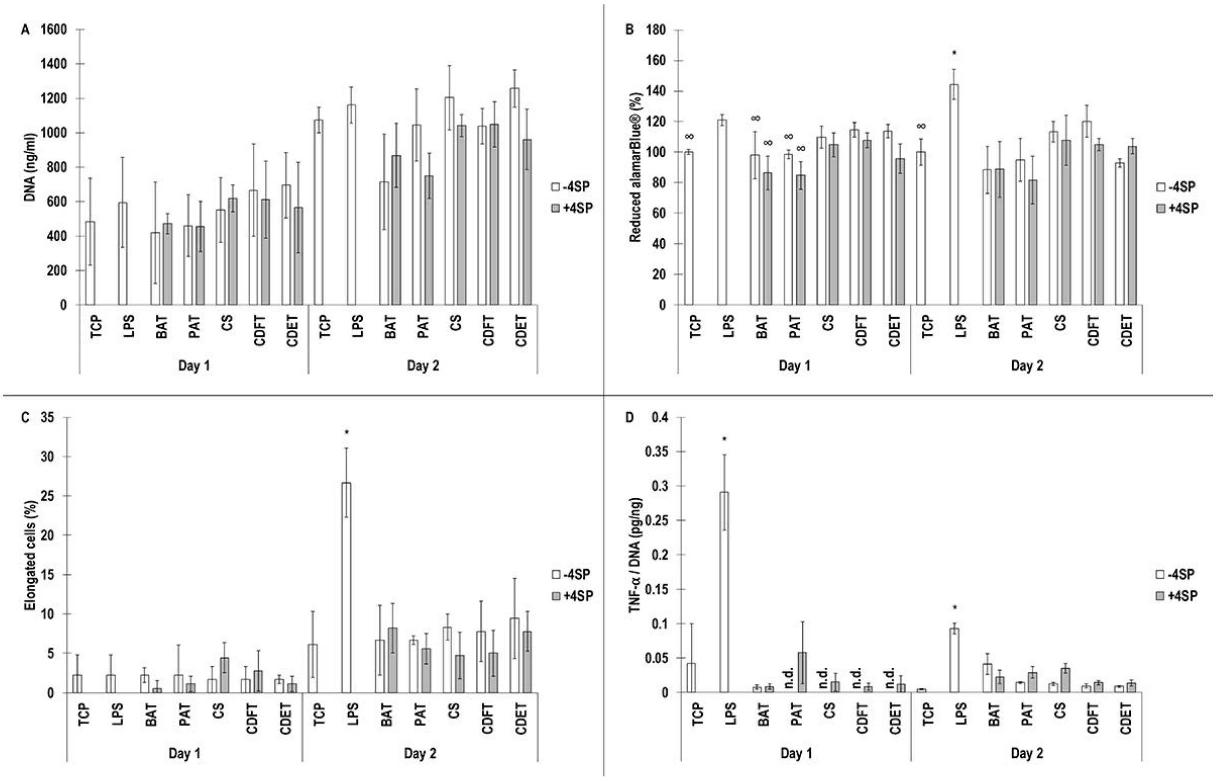

Figure S4: Rhodamine-conjugated phalloidin (red) stained cytoskeleton and 4' 6'-diamino-2-phenylindole (blue) stained nuclei of indirect human macrophage cultures for 1 and 2 days on tissue culture plastic (TCP), on lipopolysaccharide (LPS) treated TCP and on non-crosslinked (-4SP) and crosslinked (+4SP) bovine Achilles tendon (BAT), porcine Achilles tendon (PAT), caprine skin (CS), caprine digital flexor tendon (CDFT) and caprine digital extensor tendon (CDET) collagen scaffolds. #: indicates highest ($p < 0.05$) population. White arrows indicate elongated cells. N = 3.

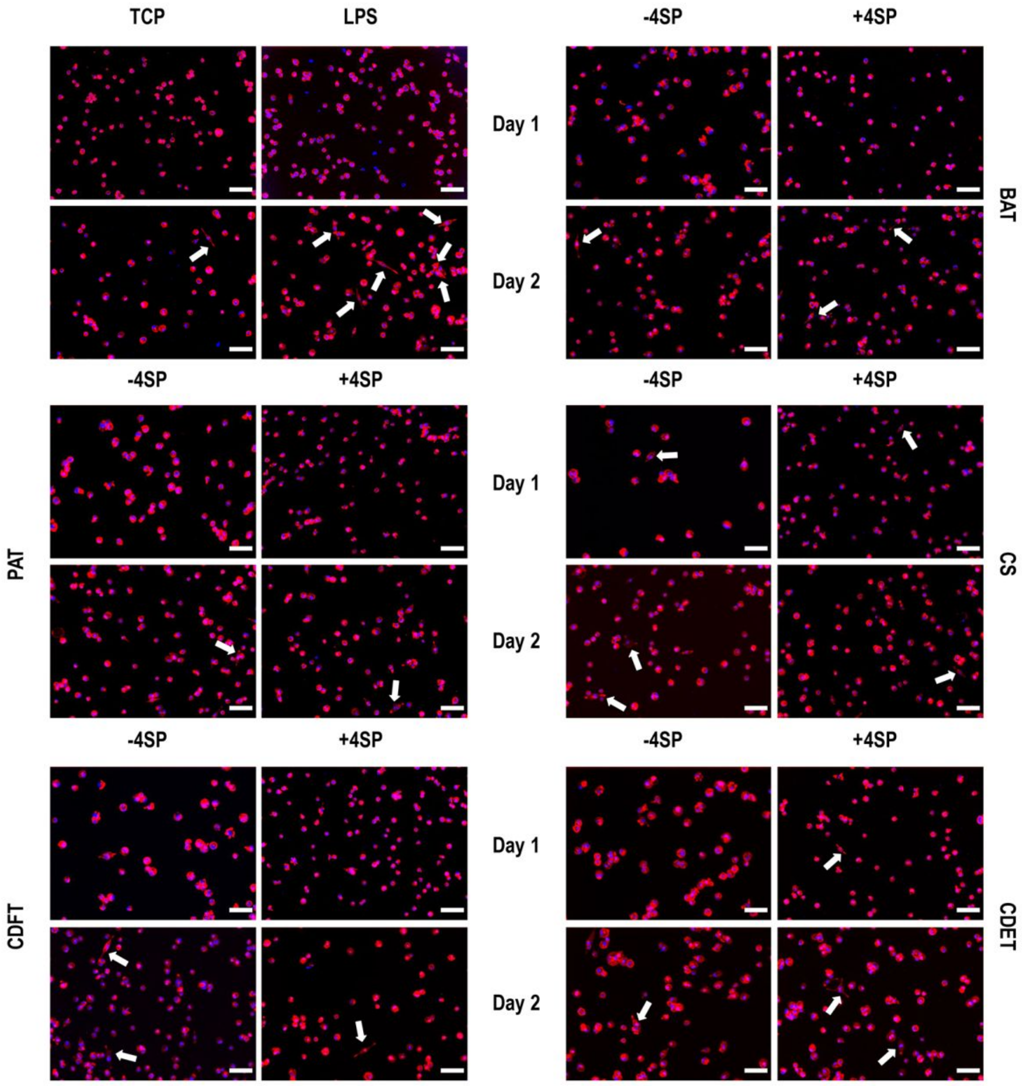

Supplement: Supplementary file 1 [file bm5c00309_si_001.pdf]
